# The Certainties and Uncertainties of the Medical Outlook

**Published:** 1919-09-06

**Authors:** 


### The Hospital

The Workers' Newspaper of Administrative Medicine and Institutional Life, Administration, National Insurance and Health.

No. 1735, Vol. LXVI. SATURDAY, SEPTEMBER 6, 1919. PRICE TWOPENCE

### THE CERTAINTIES AND UNCERTAINTIES OF THE MEDICAL OUTLOOK.

It may appear a rash thing to pretend to write with confidence of the future of anything in these days of world disturbance and unrest. To judge from appearances on the surface, no order at present standeth sure, and no institution is free from the challenging demand of the reformer. On every hand we are told that we stand at the parting of the ways, and that the future, whatever its character, is going to be something entirely different from the past. The new world, according to the prophets of the gloomy school, will be deprived of all that is attractive and stimulating and inspiring, while to the optimist the prospect is rich with the certainty of better things and brighter times. Medicine is far from escaping the general discussion. Indeed, it may be doubted whether on any occasion there has been so abounding a debate on the future form and activities of medical work, and this in reference both to internal organisation and to external relationships. On the one hand, we are told that the new science is to confer a mathematical quality and dignity on the work of the doctor, while from other quarters we learn that the medical practitioner of the future is to be bound in chains by a Government Department and held in thrall by official regulations and red tape. From the one camp is proclaimed the glad news that at long last the real function and value of medical work have been discovered; from the other are heard lamentations that liberty and initiative are about to perish from the profession.

We do not propose here to decide between the rival seers, and, indeed, we are not greatly moved by their confident anticipations. Having heard many prophets, we have no great faith in those who practise this vocation, and we think it safe to say that on this occasion, as on many previous ones, the future will prove them all to be seriously at fault. Naturally the advocates of change are prone to overstate the advantages of their pet reforms, while those who stand on the ancient ways darken the outlook of any new movement, and history tells us by many examples that in neither one case nor the other are expectations fully realised. There exist in all directions forces tending to the orderly development of social life and growth, and these in the end assert themselves to the confusion of all extremists. For a time there may be wide oscillations of the balance, but sooner or later the middle position appears, and a stable equilibrium is affirmed. This principle, we doubt not, will be illustrated in the future as in the past, and will govern the development of medicine and of the medical profession, even as it rules the conditions of the national life. Evolution certainly there will be, but not revolution. Gradual change and progress are inevitable, and are in the nature of things, but the world of medicine will not be turned upside-down. Its foundations have been tested during a long record of human experience and human need, and, though they may carry new expansions and new developments, they will rest unshaken and secure.

The confidence which we here express seems to be shared by the rising generation, if we may judge from the numbers of medical students registering at the Universities and medical schools. The war, as is well known, depressed these numbers far below the average, and for the next two or three years this limitation will express itself in a reduced number of medical practitioners. But, this period once passed, the balance will be redressed, for the new students enrolled during the current year have exceeded all records, and not a few of the more important schools have been obliged to refuse or to postpone applications for admission. Thus there is no fear that disciples will fail, and youth at all events has not been dismayed by the announcement that the end of all things is at hand. We bid the newcomers welcome, and we doubt not that they will prove themselves worthy of the traditions to which they now serve themselves heirs. They will soon learn that their inheritance is not of yesterday, that it has been created by the efforts and labours of many generations, and that the qualities which created it must be continued and repeated if its borders are to be enlarged and if its high capacities in the service of mankind are to be handed down to those who in turn will take up the task. It is no light thing to enter on the study of medicine. The work is hard and continuous, and some of its best qualities must remain with but scanty recognition and reward. Yet there is ever the consciousness of duty and responsibility and the occasion for strength and decision and courage; and out of these, for the brave and resolute of heart, the abiding sense of a full life may surely be wrought. While such material benefits as substantial fortune and public honours are hardly in the picture, no medical practitioner, however modest his

**CERTAINTIES AND UNCERTAINTIES**—(*continued*).

sphere of action, need want the stimulus of mental enterprise or the consciousness of labour for his day and generation. In such a land of promise the sincere and earnest labourer will not find a reward lacking. He will at times be chastened, but out of such experiences he may draw strength and confidence, and he may be sure that opportunities for constant growth, both in knowledge and in moral discipline, will wait upon him on every hand. The ideal is never reached, but it may always be pursued. Nowhere more than in medicine do the capacities of life rest in the hands of the man himself. His work is peculiarly personal and individual, and he may in a very large degree fashion it to his will. While achievement is never complete, it abides as an enduring stimulus and inspiration.

It must, of course, be asked in what fashion and to what extent the establishment of the Ministry of Health is likely to affect the position and prospects of the practitioner of medicine, for, obviously, this recent social and political departure is a new event, and its influence may be far-reaching. The war has quickened the national consciousness in relation to the economic and industrial value of human life and of personal health and fitness. The scrutiny of the adult male population compelled by the recruiting problem showed a state of matters which surprised and in some measure shocked the nation, and the conviction arose that here was a question of which the community must take due notice. It was realised that the policy of every man for himself and we know who takes the hindmost would no longer obtain. To be effective, it was seen, the nation must be healthy, and hence the care of the individual became to a larger extent than had previously been realised a matter of public concern. How this admitted principle can be carried into action has yet to be seen, but it may be taken as certain that its application will mean an increase of medical activities directed by public authority. There are those who would bring all these activities within the grasp of Government organisation, or would at least maintain that it is the duty of the State to provide medical advice and assistance for all citizens who care to demand them. Such a claim, however, has not yet secured public opinion, and the great majority of the profession are prepared to resist it. Yet some extension of State interference is unavoidable. The creation of the new Ministry is a recognition of this truth, and, indeed, the doctrine is hardly contested. The pressure of facts which promote this conviction may be illustrated by two references, and these indicate how, when the first step is once taken, the State is compelled to larger and larger responsibilities. Compulsory education, for example, at first sight, seems no special concern of the medical profession. But note what has followed from it. The children must go to school, but ere long it is realised that a not inconsiderable proportion of them, in consequence of bodily defects or ill-health, are unable to profit from the teaching offered to them. Hence follows the medical inspection of school children. Parents are informed of the position, but not all of them are able and willing to provide the measure of treatment required. As a mere matter of economics it is absurd for the State to provide educational facilities for children whose health makes mental training an impossibility. Thus arises the necessity for medical treatment under State direction, and if this is provided for the children of one citizen it can scarcely be refused to the children of others. In a word, compulsory education has led to a large measure of medical treatment at the expense of the community for children in attendance at the nation's elementary schools. As public money is thus involved there must needs be some measure of public control, and, at least to this extent, a certain number of doctors become servants of the State and have to act under conditions prescribed by a Government Department.

Take as a second example the National Insurance Act. This has provided for the insured what is called "ordinary medical treatment," paid for in part by public money and having responsibilities to the Government Commissioners. Here again is a first step. But events soon compel a recognition of the truth that the provision is insufficient. It does not include the expert advice needed in certain forms of illness, nor the laboratory investigations which modern medical methods to a growing extent demand, nor opportunities for the institutional treatment and nursing care so necessary for serious cases. Clearly the business cannot remain where it is. But to mend it means money, and there is no purse available but that of the State. Necessarily if the State provides money it must exercise some measure of control, so that a still larger body of doctors must be added to the list of State servants. A further step almost inevitably follows. If the expensive organisations above named are supplied out of public funds, is it possible to limit such services to insured persons? They are paid for by the general body of the citizens, and it may be not improperly urged that all citizens therefore must have access to them. In a word, we are face to face with the provision of at least certain forms of medical serviee at the public expense and available for any citizen who cares to demand them. There were those who said from the outset that the National Insurance Act would lead inevitably to a universal State Medical Service. Others believed, and believe yet, that a halting-place short of this arrangement can be found. And there for the present the matter rests. What the new entrants in the profession may realise is that opportunities for work are going to be more numerous and more varied. For those who are inclined to the certainties which are attached to public appointment there are going to be multiplied openings and chances. But the man who prefers a wider individual liberty need not be dismayed. The British public generally is not likely to discard the personal and trusted medical adviser and friend for the Government doctor, and medicine, as we believe, is not at its best when officially controlled. It invites to initiative and independence and enterprise, tempered by a sense of duty and responsibility, and those who serve it in this spirit will have no reason to regret their choice.

